# Characterization of Two Novel Intronic Variants Affecting *Splicing* in *FBN1*-Related Disorders

**DOI:** 10.3390/genes10060442

**Published:** 2019-06-10

**Authors:** Carmela Fusco, Silvia Morlino, Lucia Micale, Alessandro Ferraris, Paola Grammatico, Marco Castori

**Affiliations:** 1 Division of Medical Genetics, Fondazione IRCCS-Casa Sollievo della Sofferenza, 71013 San Giovanni Rotondo FG, Italy; l.micale@operapadrepio.it (L.M.); m.castori@operapadrepio.it (M.C.); 2 Laboratory of Medical Genetics, Department of Molecular Medicine, Sapienza University, San Camillo-Forlaninin Hospital, 00152 Rome, Italy; silvia_morlino@yahoo.it (S.M.); aferraris@scamilloforlanini.rm.it (A.F.); paola.grammatico@uniroma1.it (P.G.)

**Keywords:** fibrillin 1, Marfan syndrome, MASS syndrome, mRNA, splicing

## Abstract

*FBN1* encodes fibrillin 1, a key structural component of the extracellular matrix, and its variants are associated with a wide range of hereditary connective tissues disorders, such as Marfan syndrome (MFS) and mitral valve–aorta–skeleton–skin (MASS) syndrome. Interpretations of the genomic data and possible genotype–phenotype correlations in *FBN1* are complicated by the high rate of intronic variants of unknown significance. Here, we report two unrelated individuals with the *FBN1* deep intronic variants c.6872-24T>A and c.7571-12T>A, clinically associated with MFS and MASS syndrome, respectively. The individual carrying the c.6872-24T>A variant is positive for aortic disease. Both individuals lacked ectopia lentis. In silico analysis and subsequent mRNA study by RT-PCR demonstrated the effect of the identified variant on the splicing process in both cases. The c.6872-24T>A and c.7571-12T>A variants generate the retention of intronic nucleotides and lead to the introduction of a premature stop codon. This study enlarges the mutation spectrum of *FBN1* and points out the importance of intronic sequence analysis and the need for integrative functional studies in *FBN1* diagnostics.

## 1. Introduction

The *FBN1* gene is located in 15q21.1 and includes 65 exons which encode for fibrillin 1, a large glycoprotein constituted by 47 cysteine-rich epidermal growth factor (EGF)-like domains and seven motifs homologous to the binding protein for transforming growth factor beta (TGFβ) [1,2]. Fibrillin 1 is a major structural component of the extracellular matrix microfibrils and thus, gives stability and elasticity to many tissues [2]. Variants in *FBN1* cause a wide range of autosomal dominant heritable connective tissue disorders, including Marfan syndrome (MFS; OMIM 154700), mitral valve–aorta–skeleton–skin syndrome (MASS syndrome; OMIM 604308), Marfan lipodystrophy syndrome (OMIM 616914), isolated autosomal dominant ectopia lentis (OMIM 129600), Weill–Marchesani syndrome type 2 (OMIM 608328), acromicric dysplasia (OMIM 102370), geleophysic dysplasia type 2 (OMIM 614185), stiff skin syndrome (OMIM 184900), and autosomal dominant thoracic aortic aneurysms and dissections [3].

To date, over 1800 different *FBN1* germline variants have been identified so far (UMD-*FBN1*, http://www.umd.be/FBN1/) [4,5] and only a few genotype–phenotype correlations are available [6,7]. For example, nonsense, frameshift, and some splicing variants appear correlated with a more severe skin and skeletal phenotype, as compared to in-frame variants [8]. Other studies suggest that variants affecting or creating cysteine residues are more commonly associated with ectopia lentis, while *null* alleles typically combine with an increased rate of aortic events in young age and thoracic deformities [7]. In the suspicion of a *FBN1*-related disorder, the identification of the causative variant is not only relevant for diagnosis confirmation and genetic counseling but is also increasingly useful for personalized medicine approaches, comprising preventive drug treatment and aortic surgery planning [9].

The introduction of next generation sequencing (NGS) technologies in molecular diagnostics improved turnaround time and costs but did not significantly affect the clinical interpretation of variants of uncertain significance, such as intronic nucleotide changes which potentially affect splicing. The burden associated with uncertain results related to these variants is still high in MFS and related disorders, as point variants possibly impacting FBN1 pre-mRNA splicing seem to account for ~10% of reported molecular findings in MFS [10]. In these cases, optimal clinical interpretation should integrate molecular findings with customized studies exploring variant effects at the transcriptional or translational levels.

Here, we report two unrelated individuals with MFS and MASS syndrome, respectively, which were associated with different intronic variants in *FBN1*. Characterization of the effects of these variants on mRNA allowed us to support their pathogenicity and, therefore, to use them for patient and family management.

## 2. Materials and Methods

All investigated subjects signed informed consent for molecular testing and research use of biological and related clinical data. Molecular testing on cDNA and mRNA in individual 1 and 2 were carried out within the routine clinical diagnostic activities of the Division of Medical Genetics of Foundation IRCCS-Casa Sollievo della Sofferenza, San Giovanni Rotondo (Italy). The results of this work are part of a larger research project approved by our local ethics committee (approval protocol no. GTB12001). 

### 2.1. Clinical Description: Individual 1

This was a 27-year-old man, the second child of unrelated parents. His father was 50-year-old, 191 cm tall and affected by dilatation of the aortic root and ascending aorta, which required surgery at 45 years. The mother and sister were healthy, and 172 and 161 cm tall, respectively. The proband was born at term after an uneventful pregnancy. At birth, he presented respiratory distress, which needed intensive care support for a few days. The neonatal period was otherwise normal, as well as psychomotor development, scholarship, and mentation. At 11 years, progressive scoliosis required an orthopedic corset for some years. The patient suffered from recurrent spontaneous pneumothorax with four episodes between 12 and 14 years of age. For this, he successfully underwent pleurodesis at 14 years. At 18 years, the previous diagnosis of aortic disease in the father prompted full cardiologic assessment in the proband who showed dilatation of the aortic root and mitral valve prolapse. Since then, he was under preventive pharmacologic therapy. Current treatment includes losartan 50 mg/day (single dose) and nebivolol 50 mg/day (single dose), without significant side effects. The last heart ultrasound, at 27 years, showed a stable dilatation of the aortic root (48 mm; Z score 171.13 = 4.66 SD [11]) with normal ascending aorta (34 mm), minimal insufficiency of the aortic valve, prolapse of the anterior mitral leaflet with minimal insufficiency, and mild insufficiency of the tricuspid valve. Dilatation of the aortic root (48 × 43 mm) was also confirmed by angio-computer tomography. A total spine MRI at 23 years showed bilateral dural ectasias of the lumbar spine. A recent ophthalmologic exam excluded lens dislocation and revealed mild myopia (−2 diopters on the right and −1.5 diopters on the left).

**Figure 1:**
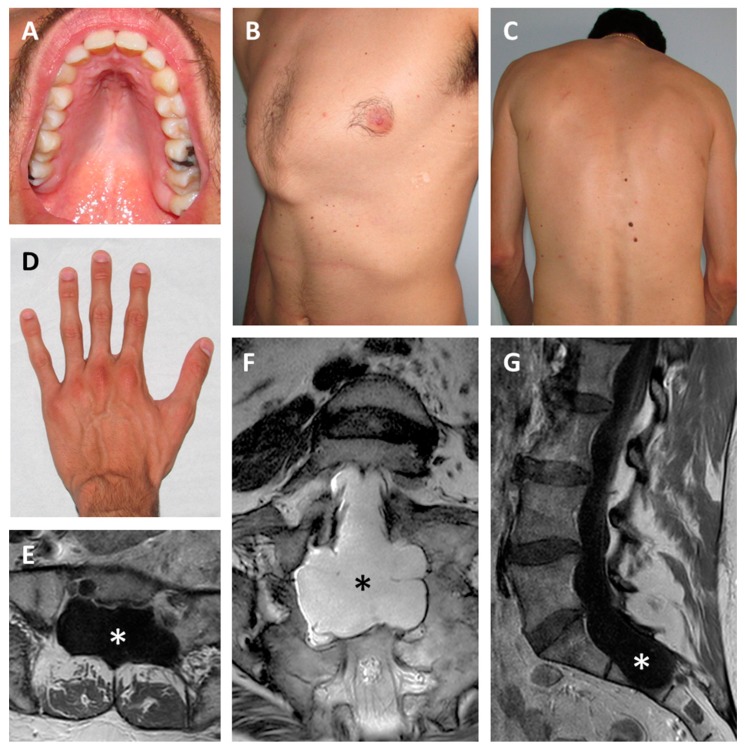
Clinical features. Individual 1 showing high-arched palate (**A**), pectus carinatum (**B**), scoliosis (**C**) and arachnodactyly (**D**). Lumbar spine MRI of Individual 2 demonstrating bilateral dural ectasias at axial (**E**), coronal (**F**), and sagittal (**G**) views. Asterisks indicate the dilated dural sac.

Physical examination included height 196 cm, weight 72 kg, arm span 198 cm, arm span/height ratio 1.01 (normal), bilateral positive wrist sign, bilateral negative thumb sign, apparent enophthalmos, down slanting palpebral fissures, malar hypoplasia, high-arched palate, severe scoliosis, pectus carinatum, bilateral valgus deformity of the elbow, bilateral metatarsus varus, some mildly atrophic post-surgical scars of the thorax, and striae rubrae of the back (Figure 1A–D). The combination of aortic root dilatation (Z-score > 2 SD) and 10 points of systemic features lead to the clinical suspect of MFS.

### 2.2. Clinical Description: Individual 2

Individual 2 was a 48-year-old, nullipara woman, the seventh child of a sibship of eleven. All brothers and sisters were healthy, but two brothers were described with tall stature (~194 cm). The mother was 165 cm tall and died at 65 years due to rupture of a cerebral aneurysm. The father was 175 tall and died at 78 years due to a stroke. Family history was negative for aortic disease. The proband was sent to clinical genetics assessment by the neurosurgeon, who first evaluated the woman for chronic back pain, diffuse spondylosis, and marked dural ectasias of the lumbar spine (Figure 1E–G). Relevant additional clinical features included moderately severe scoliosis since her teens, severe myopia (−6 diopters on the right and −8 dipters on the left), and chronic fatigue. A heart ultrasound repeatedly showed normal aortic diameters, prolapse, and mild insufficiency of the mitral valve. At 47 years, aortic root diameter was 36 mm (Z score 1.26 SD [11]). Routine ophthalmologic assessments always excluded lens dislocations. Physical examination showed height 181 cm, weight 80 kg, arm span 188 cm, arm span/height ratio 1.038 (normal), bilaterally positive wrist and thumb signs, hypermobility of fingers (but the Beighton score was 0/9), elbow limitation on both sides, enophthalmos, down slanting palpebral fissures, retrognathia, high-arched palate, striae distensae of the shoulders. The presence of 10 points of systemic features and an aortic root diameter <2 SD suggested a diagnosis of the MASS syndrome. Positive family history for tall stature and absence of aortic disease in relatives were in accordance with the diagnosis. Molecular testing in Individual 2 was carried out with a multigene panel approach including extended intronic regions around exon–intron junctions (see below).

### 2.3. Genomic DNA Extraction

Genomic DNA was extracted from individuals’ peripheral bloods by using Bio Robot EZ1 (Qiagen, Hilden, Germany), according to standard procedures. The DNA was quantified with Nanodrop 2000 C spectrophotometer (Thermo Fisher Scientific, Waltham, MA, USA).

### 2.4. Genetic Testing (Sanger Sequencing): Individual 1

Molecular analysis for individual 1 was performed by Sanger sequencing. Primers were designed using the Primer 3 Output program (http://frodo.wi.mit.edu/primer3/) to amplify extended intronic sequences and exon–intron flanking sequences of *FBN1* (RefSeq NM_000138). Primers were checked both by BLAST and BLAT against the human genome to ensure specificity. Amplified products were subsequently purified and sequenced with a ready reaction kit (BigDye Terminator v1.1 Cycle, Applied Biosystems, Foster City, CA, USA). Fragments were then purified using DyeEx plates (Qiagen, Hilden, Germany) and resolved on an automated sequencer (3130xl Genetyc analyzer DNA Analyzer, ABI Prism, Foster City, CA, USA). Sequences were analyzed using the Sequencer software (Gene Codes, Ann Arbor, MI, USA). To investigate familial segregation, both parents were sequenced for the identified variant.

### 2.5. Genetic Testing (Next-Generation Sequencing): Individual 2

Individual 2’s DNA underwent sequencing with a HaloPlex gene panel (Agilent Technologies, Santa Clara, CA, USA) designed to selectively capture known genes associated with syndromic and non-syndromic thoracic aneurysms and/or Mafanoid habitus, including: *ACTA2* (NM_001141945), *BGN* (NM_001711.5), *CBS* (NM_000071.2), *COL3A1* (NM_000090), *COL4A1* (NM_001303110), *COL5A1* (NM_000093), *COL5A2* (NM_000393), *DLG4* (NM_001365.4), *EFEMP2* (NM_016938), *ELN* (NM_000501), *EMILIN* (NM_007046.3), *FBN1* (NM_000138), *FBN2* (NM_001999), *FLNA* (NM_001110556), *FOXE3* (NM_012186), *GATA5* (NM_080473), *LOX* (NM_002317), *MAT2A* (NM_005911), *LTBP3* (NM_001130144.2), *MAT2A* (NM_005911.5), *MED12* (NM_005120.2), *MFAP5* (NM_003480), *MYH11* (NM_001040113), *MYLK* (NM_053025), *NOTCH1* (NM_017617), *PLOD1* (NM_001316320), *PRKG1* (NM_006258), *SKI* (NM_003036), *SLC2A10* (NM_030777), *SMAD2* (NM_005901.5), *SMAD3* (NM_005902), SMAD4 (NM_005359), *TAB2* (NM_015093), *TGFB2* (NM_001135599), *TGFB3* (NM_003239), *TGFBR1* (NM_001306210), *TGFBR2* (NM_001024847), *UPF3B* (NM_080632.2), and *ZDHHC9* (NM_016032.3). Targeted fragments were then sequenced on a MiSeq platform (Illumina, San Diego, CA, USA) using a MiSeq Reagent kit V3 300 cycles flow cell. Data analysis was performed considering the frequency, impact on the encoded protein, conservation, and expression of variants using distinct tools, as appropriate ANNOVAR (http://annovar.openbioinformatics.org/en/latest/), dbSNP (https://www.ncbi.nlm.nih.gov/snp), 1000 Genomes (http://www.internationalgenome.org), ExAC (http://exac.broadinstitute.org). Selected variants are interpreted according to the American College of Medical Genetics and Genomics/Association for Molecular Pathology (ACMGG/AMP) [12]. The identified variant was confirmed by Sanger sequencing. We checked the selected variant in the UMD-*FBN1* database, a computerized database that currently contains information about the published mutations of the *FBN1* gene.

### 2.6. Variant Designation

Nucleotide variant nomenclature follows the format indicated in the Human Genome Variation Society (HGVS, http://www.hgvs.org) recommendations. DNA variant numbering system refers to cDNA. Nucleotide numbering uses +1 as the A of the ATG translation initiation codon in the reference sequence, with the initiation codon as codon 1.

### 2.7. In Silico Prediction

In silico analysis of identified intronic variants was conducted by running three independent algorithms for splice signal detection: NetGene2 (http://www.cbs.dtu.dk/services/NetGene2/), Berkeley Drosophila Genome Project (BDGP, http://www.fruitfly.org/seq_tools/splice.html), and Human Splicing Finder (HSF, http://www.umd.be/HSF3/).

### 2.8. RNA Extraction and Reverse Transcription–Polymerase Chain Reaction

Total RNA was extracted from lymphocytes fraction isolated from peripheral blood. Total RNA was extracted using RNeasy Mini Kit (Qiagen, Hilden, Germany), treated with DNase-RNase free (Qiagen, Hilden, Germany), quantified by Nanodrop (Thermo Fisher Scientific, Waltham, MA, USA), and reverse-transcribed using QuantiTect Reverse Transcription Kit (Qiagen, Hilden, Germany) according to the manufacturer’s instructions. Primers used for DNA amplification and reverse transcription–polymerase chain reaction are reported in Table 1.

## 3. Results

### 3.1. Individual 1

**Figure 2:**
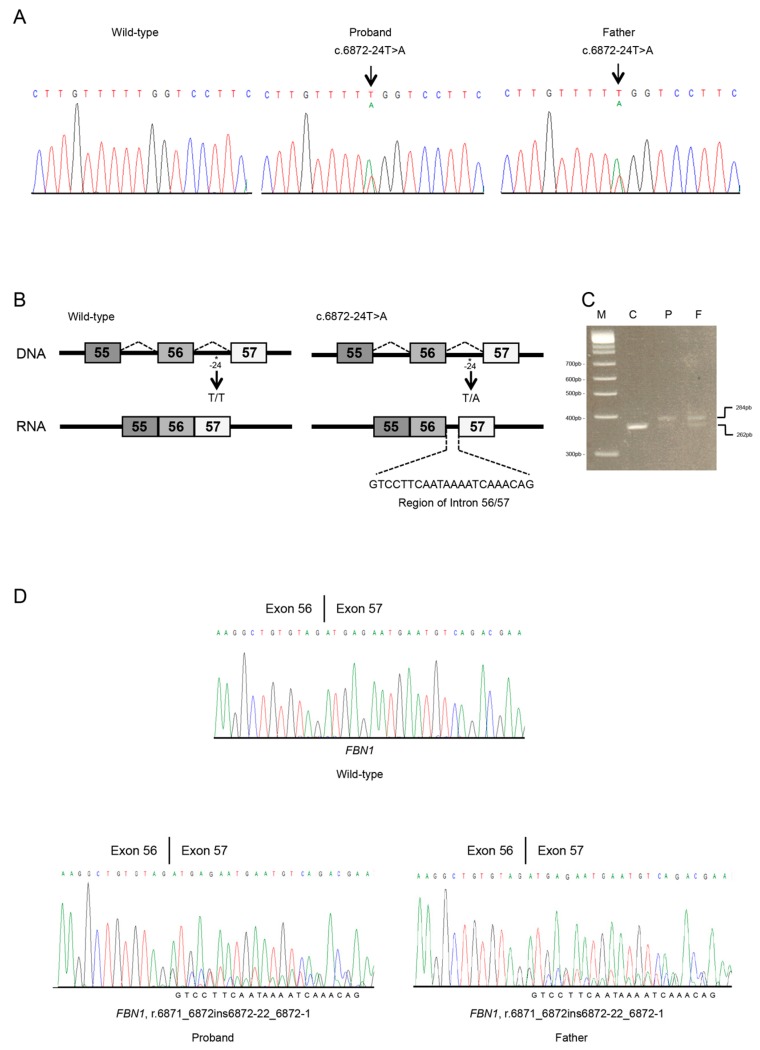
Molecular analyses of individual 1. (**A**) Sanger sequencing in an unaffected (unrelated) control (CTRL), the proband and his father of the *FBN1* region spanning the 56 to 57 exon–intron junction. The *FBN1* genomic variant is indicated by an arrow. (**B**) *S*chematic representation of *FBN1* region including 55 to 57 exons and introns. Rectangles represent exons; thin horizontal lines represent introns. The position of the identified variant is indicated with an asterisk. Wild type and abnormal transcripts generated by the c.6872-24T>A splicing variant are shown. (**C**) Total RNA of a normal (unrelated) control, the proband, and his father were used for RT-PCR of the *FBN1* transcript. The same amounts of patient’s and control cDNA were PCR amplified. PCR products were analyzed by electrophoresis on 3.5% agarose gel. M, DNA marker; C, control; P, proband; F, father. Affected individuals (lane P and F) present both the wild-type mRNA (lower band) and aberrantly spliced transcript (upper band). (**D**) Sanger sequencing of the *FBN1* transcript, including exons 56 to 57 in an unaffected (unrelated) control (C), the proband (P), and his father (F). Variant position on mRNA level was annotated (r.6871_6872ins6872-22_6872-1). The intronic region was reported at the bottom of the Sanger chromatograms of all affected individuals.

Molecular testing was carried out on the Individual 1 DNA by Sanger sequencing of the *FBN1* gene, and this gave normal results. Multiple ligation-dependent probe amplification analysis for *FBN1* intragenic deletions/insertions resulted normal. Given the convincing clinical picture and positive family history, re-analyzing raw data from the Sanger sequence of extended intronic sequences was carried out and revealed a novel *FBN1* heterozygous splicing variant c.6872-24T>A, p.(?) (Figure 2A). Co-segregation with the disease was demonstrated on the father’s DNA.

Computational predictions conducted using NetGene2, BDGP, and HSF revealed that the intronic c.6872-24T>A variant might influence the splicing process by differentially affecting canonical versus cryptic splice site utilization. The newly detected variant was predicted to insert 22 nucleotides (nt) of intron 56 into the mature mRNA (Figure 2B). This should give rise to frameshift and a PTC (p.(Asp2291Glyfs*9)), leading to the loss of the TGFβ binding protein homologous motifs, the last seven cysteine-rich EGF-like domains, and the FibuCTDIII-like motif.

The effect of the *FBN1* splice site variant c.6872-24T>A at the mRNA level was ascertained by in vitro RT-PCR amplification of *FBN1* RNA of Individual 1 and his father. The sequencing of the RT-PCR products spanning the 55 to 57 exons of *FBN1* showed the insertion of 22 nts of intron 56–57 in the *FBN1* mRNA of Individual 1 and his father (r.6871_6872ins6872-22_6872-1) (Figure 2C,D). Electropherogram showed a significant reduction of the peaks corresponding to the mutated allele. The aberrantly spliced transcript is not present in the expressed sequence tag database (EST, https://www.ncbi.nlm.nih.gov/dbEST/index.html) of *FBN1*. Following available standards for the interpretation of sequence variants [12], the *FBN1* c.6872-24T>A variant was classified as likely pathogenic and submitted to the Leiden Open Variation Database (LOVD, https://www.lovd.nl; accession number: # 00229824).

### 3.2. Individual 2

**Figure 3:**
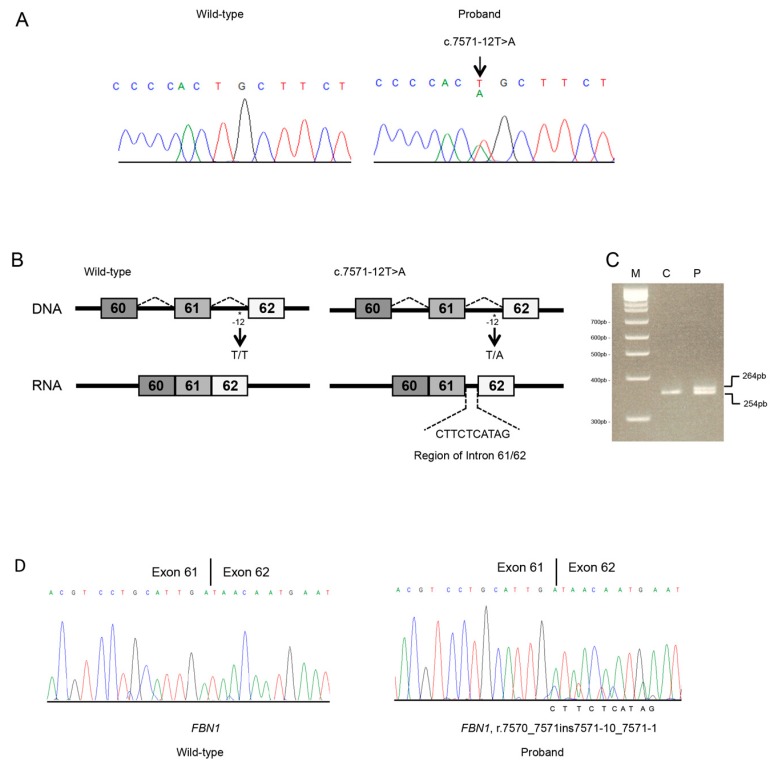
Molecular analyses of individual 2. (**A**) Sanger sequencing in an unaffected (unrelated) control and the proband of the *FBN1* region spanning 61 to 62 exon–intron junction. The *FBN1* genomic variant is indicated by an arrow. (**B**) *S*chematic representation of *FBN1* region including 61 to 62 exons and introns. Rectangles represent exons; thin horizontal lines represent introns. The position of the identified variant is indicated with an asterisk. Abnormal transcripts generated by the c.7571-12T>A splicing variant. (**C**) Total RNA of a normal (unrelated) control and the proband were used for RT-PCR of the *FBN1* transcript. The same amounts of patient and control cDNA were PCR amplified. PCR products were analyzed by electrophoresis on 3.5% agarose gel. M, DNA marker; C, control; P, the proband. M, DNA marker; C, control; P, proband; F, father. Affected individual (lane P) presents both wild-type (lower band) and aberrant transcript (upper band). (**D**) Sanger sequencing of the *FBN1* transcript, including exons 61 to 62 in an unaffected (unrelated) control (C) and the proband (P). Variant position on mRNA level was annotated (r.7570_7571ins7571-10_7571-1). The intronic region was reported at the bottom of the Sanger chromatogram of the affected individual.

Targeted NGS analysis revealed the *FBN1* heterozygous c.7571-12T>A variant in intron 61 (Figure 3A). No other candidate variant was found in the remaining genes. The c.7571-12T>A variant was not reported in the UMD-*FBN1* and was absent in the proband’s healthy sister. Both parents died, and the other brothers and sisters were not available for genetic testing.

The splicing predictor tools showed that the variant might alter the splicing process. In particular, all predicted with high probability that the c.7571-12T>A intronic variant activates a cryptic acceptor splice site within intron 61 that, in turn, leads to an inefficient recognition of the canonical splice site (Figure 3B) and insertion of intronic nucleotides in the *FBN1* mRNA. Sequence analysis on cDNA confirmed the c.7571-12T>A variant causes a shift from canonical toward cryptic splicing that leads to the inclusion of 10 nts of the 61 to 62 intronic sequence (r.7570_7571ins7571-10_7571-1) (Figure 3C,D) and a frameshift which generate a PTC in exon 61 (p.(Asp2524Argfs*5)). The truncated protein loses the last seven cysteine-rich EGF-like domains and the FibuCTDIII-like motif. Electropherogram showed a reduction of the peaks corresponding to the mutated allele. The sequence of the alternative mRNA was absent in the EST database. The *FBN1* c.7571-12T>A variant was therefore interpreted as likely pathogenic and submitted to LOVD (accession number: #00229832).

## 4. Discussion

In this work, we added two novel intronic variants to the *FBN1* mutational repertoire and demonstrated their effect at the mRNA level. Our findings highlight the impact of variants affecting non-coding DNA in MFS and related disorders, and the opportunity to re-consider the standard diagnostic approaches, at least, in specific cases. In particular, molecular studies revealed that the novel c.6872-24T>A and c.7571-12T>A *FBN1* variants induce aberrant pre-mRNA splicing by the generation of a new cryptic acceptor site that outcompetes the canonical splice site and generate exonization of intronic sequences. This, in turn, results in a frameshift with the introduction of a PTC. In both cases, PTC is predicted to produce a truncated fibrillin 1 with a loss of critical C-terminal domains of fibrillin 1 [13].

The mechanism(s) by which *FBN1* splicing variants might exert their pathogenic effect is (are) complex, and both dominant negative and loss-of-function effects have been proposed based on the predicted consequences on mRNA, and protein structure and function(s) [14,15,16]. In splicing variants generating out-of-frame deletions/insertions, the introduction of a PTC and the synthesis of a truncated protein are assumed. However, PTC might elicit nonsense-mediated mRNA decay (NMD), with a variable proportion of the mutated allele acting as a *null* allele. If NMD affects ~100% of the transcripts, haploinsufficiency is the leading molecular pathogenesis. Conversely, the production of a truncated fibrillin 1 due to complete NMD escape, can impact its function due to truncated protein production. In proteins with pleiotropic effects and distinct domains with different functions, such as fibrillin 1 [3], the length of the synthesized truncated protein and the differential expression of the gene in the different tissues are the leading modulator factors of its residual functions. A mixed pathogenesis should also be considered for PTC that allow only in part NMD. In splicing variants leading to in-frame deletions/insertions, the predicted consequence might mirror the dominant negative effect attributed to point variants [17].

Literature on *FBN1* intronic variants affecting non-canonical splice sites is currently limited to single reports, and the emerging genotype–phenotype correlations are purely speculative. For example, Wang et al., by reporting a small case series of familial aortic aneurysms and dissection, studied the effect of the *FBN1* c.5917+6T>C variant in HEK293 cells and concluded that skipping of exon 47 seems to associate with a higher rate of cardiovascular involvement [18]. Wypasek and coworkers reported an adult with MFS, severe cardiac involvement but normally placed lens, and the *FBN1* c.1589-9T>A affecting splicing by in silico and in vitro investigations [19]. Recently, a further MFS patient with full cardiac, ocular, and skeletal phenotype has been reported with the *FBN1* c.2678-15C>A variant affecting splicing by minigene approaches [20].

In our study, both variants are predicted to result in a truncated protein. Although we did not further investigate their effect, both transcripts are likely destined, at least in part, to NMD, as the PTC falls in more than 50 nts upstream of the last exon–exon junction within the mRNA [21]. This might be hypothesized by the differences in peak heights in the electropherograms of *FBN1* transcripts (Figure 2 and Figure 3). This result suggests that a portion of aberrantly *FBN1*-spliced transcripts could be degraded by NMD process in both cases. *FBN1* haploinsufficiency due to NMD-mediated degradation of aberrant mRNA might be the leading candidate molecular mechanism causing disease for the novel c.6872-24T>A and c.7571-12T>A variants. However, we cannot exclude that a proportion of the mutated transcripts escapes NMD at least in blood cells. Therefore, both NMD and the synthesis of a truncated protein may result from the identified causative variants. In Individual 1 the predicted truncated protein (p.(Asp2291Glyfs*9)) losses the seven TGFβ binding protein homologous motifs, the last seven cysteine-rich EGF-like domains, and the FibuCTDIII-like motif; while in Individual 2, protein truncation should occur later (p.(Asp2524Argfs*5)) preserving the loss of TGFβ binding protein homologous motifs. Individual 1 (and his affected father) showed severe aortic disease, a feature that was not observed in Individual 2, and both individuals lack ectopia lentis.

The reasons underlying the different clinical diagnoses in these two patients are hard to define. We can speculate that in Individual 1 aortic involvement might be, at least in part, due to the loss of TGFβ binding domain, which, in turn, is preserved in the truncated protein predicted in Individual 2 (MASS syndrome), who showed normal aorta. In fact, perturbed binding with TGFβ results in the alteration of elasticity and microfibrils stability, two factors primary influencing aortic wall integrity [22]. A trend toward an increased risk for ectopia lentis was reported in cysteine-destroying or cysteine-creating variants [2,15], a mechanism not easily evoked in our two Individuals. Therefore, our observation testifies once more the difficulties in tracing consistent genotype–phenotype correlations in MFS and the need for international collaborations working towards this aim.

In this work, we highlighted the importance of including extended intronic regions in the analysis of *FBN1* for clinical purposes. This remains valid in the era of NGS and should be considered at the stage of (virtual) multigene panel design, as well as during variant prioritization and post-analytical phase. Such an approach also needs the availability of additional tests validated for diagnostics and able to explore variant effects at the mRNA or post-transcriptional levels for supporting the clinical interpretation of the genomic data. Therefore, this work points out the need for introducing functional investigations in the diagnostic workflow of at least selected disorders or gene panels, for which a high rate of intronic variants with a potential pathogenic effect is expected. The description of further individuals with splicing variants with documented effects at the various post-genomic levels seems critical for improving our knowledge on this issue.

## Figures and Tables

**Table 1:** Primers used for DNA amplification reverse transcription of *FBN1* fragments carrying the investigated variants (RefSeq NM_000138) in individual 1 and individual 2.

Individual	Primer	Sequence	Amplicon Size (pb)	Application
Individual 1	FBN1_Int56-57_F	TTTTGAGCCATGTGAACAGATT	320	DNA
	FBN1_EX57_R	AAACCCATCATTACACTCACAGG		
	FBN1_EX55_F	ATATGTGCTCAGAGAAGACCGTA	262	cDNA
	FBN1_EX57_R	AAACCCATCATTACACTCACAGG		
Individual 2	FBN1_Int61-62_F	TCCGAGTTATCCTTCTAATTTTCT	313	DNA
	FBN1_EX62_R	TATCTCATAGAGGCTGATGATGAAG		
	FBN1_EX61_F	GCAACCAAGCAACACAACTG	254	cDNA
	FBN1_EX63_R	TTACCCTCACACTCGTCCAC		

## References

[1] Ramirez F., Dietz H.C. (2007). Fibrillin-rich microfibrils: Structural determinants of morphogenetic and homeostatic events. J. Cell Physiol..

[2] Verstraeten A., Alaerts M., Van Laer L., Loeys B. (2016). Marfan Syndrome and Related Disorders: 25 Years of Gene Discovery. Hum. Mutat..

[3] Sakai L.Y., Keene D.R. (2018). Fibrillin protein pleiotropy: Acromelic dysplasias. Matrix Biol.

[4] Collod-Beroud G., Le Bourdelles S., Ades L., Ala-Kokko L., Booms P., Boxer M., Child A., Comeglio P., De Paepe A., Hyland J.C. (2003). Update of the UMD-FBN1 mutation database and creation of an FBN1 polymorphism database. Hum. Mutat..

[5] Frederic M.Y., Lalande M., Boileau C., Hamroun D., Claustres M., Beroud C., Collod-Beroud G. (2009). UMD-predictor, a new prediction tool for nucleotide substitution pathogenicity-application to four genes: FBN1, FBN2, TGFBR1, and TGFBR2. Hum. Mutat..

[6] Faivre L., Collod-Beroud G., Callewaert B., Child A., Binquet C., Gautier E., Loeys B.L., Arbustini E., Mayer K., Arslan-Kirchner M. (2009). Clinical and mutation-type analysis from an international series of 198 probands with a pathogenic FBN1 exons 24-32 mutation. Eur. J. Hum. Genet..

[7] Aubart M., Gazal S., Arnaud P., Benarroch L., Gross M.S., Buratti J., Boland A., Meyer V., Zouali H., Hanna N. (2018). Association of modifiers and other genetic factors explain Marfan syndrome clinical variability. Eur. J. Hum. Genet..

[8] Ergoren M.C., Turkgenc B., Terali K., Rodoplu O., Verstraeten A., Van Laer L., Mocan G., Loeys B., Tetik O., Temel S.G. (2019). Identification and characterization of a novel FBN1 gene variant in an extended family with variable clinical phenotype of Marfan syndrome. Connect. Tissue Res..

[9] Wagner A.H., Zaradzki M., Arif R., Remes A., Muller O.J., Kallenbach K. (2019). Marfan syndrome: A therapeutic challenge for long-term care. Biochem. Pharmacol..

[10] Zeyer K.A., Reinhardt D.P. (2015). Engineered mutations in fibrillin-1 leading to Marfan syndrome act at the protein, cellular and organismal levels. Mutat. Res. Rev. Mutat. Res..

[11] Campens L., Renard M., Callewaert B., Coucke P., De Backer J., De Paepe A. (2013). New insights into the molecular diagnosis and management of heritable thoracic aortic aneurysms and dissections. Pol. Arch. Med. Wewn..

[12] Richards S., Aziz N., Bale S., Bick D., Das S., Gastier-Foster J., Grody W.W., Hegde M., Lyon E., Spector E. (2015). Standards and guidelines for the interpretation of sequence variants: a joint consensus recommendation of the American College of Medical Genetics and Genomics and the Association for Molecular Pathology. Genet. Med..

[13] Jensen S.A., Aspinall G., Handford P.A. (2014). C-terminal propeptide is required for fibrillin-1 secretion and blocks premature assembly through linkage to domains cbEGF41-43. Proc. Natl. Acad. Sci. USA.

[14] Matyas G., Alonso S., Patrignani A., Marti M., Arnold E., Magyar I., Henggeler C., Carrel T., Steinmann B., Berger W. (2007). Large genomic fibrillin-1 (FBN1) gene deletions provide evidence for true haploinsufficiency in Marfan syndrome. Hum. Genet..

[15] Judge D.P., Biery N.J., Keene D.R., Geubtner J., Myers L., Huso D.L., Sakai L.Y., Dietz H.C. (2004). Evidence for a critical contribution of haploinsufficiency in the complex pathogenesis of Marfan syndrome. J. Clin. Investig..

[16] Takeda N., Inuzuka R., Maemura S., Morita H., Nawata K., Fujita D., Taniguchi Y., Yamauchi H., Yagi H., Kato M. (2018). Impact of Pathogenic FBN1 Variant Types on the Progression of Aortic Disease in Patients With Marfan Syndrome. Circ. Genom. Precis. Med..

[17] Dietz H.C., Valle D., Francomano C.A., Kendzior R.J., Pyeritz R.E., Cutting G.R. (1993). The skipping of constitutive exons in vivo induced by nonsense mutations. Science.

[18] Wang W.J., Han P., Zheng J., Hu F.Y., Zhu Y., Xie J.S., Guo J., Zhang Z., Dong J., Zheng G.Y. (2013). Exon 47 skipping of fibrillin-1 leads preferentially to cardiovascular defects in patients with thoracic aortic aneurysms and dissections. J. Mol. Med..

[19] Wypasek E., Potaczek D.P., Hydzik M., Stapor R., Raczkowska-Muraszko M., Weiss J., Maugeri A., Undas A. (2018). Detection and a functional characterization of the novel FBN1 intronic mutation underlying Marfan syndrome: Case presentation. Clin. Chem. Lab. Med..

[20] Torrado M., Maneiro E., Trujillo-Quintero J.P., Evangelista A., Mikhailov A.T., Monserrat L. (2018). A Novel Heterozygous Intronic Mutation in the FBN1 Gene Contributes to FBN1 RNA Missplicing Events in the Marfan Syndrome. BioMed Res. Int..

[21] Isken O., Maquat L.E. (2007). Quality control of eukaryotic mRNA: safeguarding cells from abnormal mRNA function. Genes Dev..

[22] Le Goff C., Mahaut C., Wang L.W., Allali S., Abhyankar A., Jensen S., Zylberberg L., Collod-Beroud G., Bonnet D., Alanay Y. (2011). Mutations in the TGFbeta binding-protein-like domain 5 of FBN1 are responsible for acromicric and geleophysic dysplasias. Am. J. Hum. Genet..

